# Incidence of opioid-induced constipation in non-cancer patients using weak opioids for chronic pain in Japan: a cohort study

**DOI:** 10.1038/s41598-025-01770-0

**Published:** 2025-05-19

**Authors:** Akira Hashimoto, Yasuhide Morioka, Shihomi Wada, Yuichi Koretaka, Motoki Sonohata

**Affiliations:** 1 Department of Orthopaedic Surgery, Japan Community Health Care Organization (JCHO) Saga Central Hospital, 3-8-1, Hyogominami, Saga City, Saga 849-8522 Japan; 2 Medical Affairs Department, Shionogi & Co., Ltd., Nissay Yodoyabashi East, 3-13, Imabashi 3-chome, Chuo-ku, Osaka, 541-0042 Japan; 3 Data Science Department, Shionogi & Co., Ltd., 4F MTR building, 3-6-3, Awajimachi, Chuo-ku, Osaka, 541-0047 Japan

**Keywords:** Japan, Non-cancer pain, Opioid-induced constipation, Rome IV criteria, Weak opioids, Chronic pain, Constipation, Outcomes research

## Abstract

Opioid-induced constipation (OIC) is a common adverse drug reaction of opioids. However, limited evidence exists regarding the incidence of OIC, as diagnosed using the Rome IV criteria, in non-cancer patients newly prescribed weak opioids for chronic pain in Japan. This study aimed to clarify the incidence of OIC in patients aged ≥ 18 years who were newly prescribed weak opioids in outpatient settings in Japan. This study was conducted from February 1, 2023, to July 13, 2024 (trial registration number: UMIN000050203), using a patient-reported, web-based questionnaire survey. Of the 64 participants (mean age: 53.2 years) included, 63 participants received weak opioids for ≥ 7 days. The cumulative incidence of OIC (95% confidence interval) was 30.2% (20.4–43.1) in the first week and increased to 49.2% (37.7–62.1) by the end of the second week. Common symptoms included straining (66.7%), incomplete evacuation (49.2%), and hard stools (22.2%). Although the results were derived from exploratory analyses, no risk factors for the development of OIC were identified among participants’ demographics, clinical characteristics (including pain sites), or the weak opioid usage and dosage. These findings emphasize the need for caution regarding the onset of OIC when initiating pain management with weak opioids.

**Supplementary Information:**

The online version contains supplementary material available at 10.1038/s41598-025-01770-0.

## Introduction

Weak opioids are commonly used in managing chronic non-cancer pain in Japan^1–3^. Nonetheless, they are associated with gastrointestinal adverse drug reactions such as nausea, vomiting, and opioid-induced constipation (OIC)^1,4^. The presence of opioid receptors within the enteric nervous system, which act as binding sites for opioid molecules, leads to OIC, a dose-independent adverse drug reaction^1,4–7^.

A recent web-based survey conducted in Japan revealed that approximately 30% of patients with chronic non-cancer pain who were using either weak or strong opioid analgesics experienced OIC, as diagnosed according to the Rome IV criteria^1^. Symptoms of OIC commonly include firm, difficult, or infrequent passage of hard stools, accompanied by straining during defecation and feelings of incomplete evacuation or anorectal obstruction^6,8^. For instance, a longitudinal, cross-sectional survey conducted by Coyne et al. involving patients receiving daily opioid therapy for more than 4 weeks found that a majority of patients (approximately 69%) experienced common symptoms of constipation, such as straining, bowel movements too hard, flatulence, and bloating, leading to impaired daily activities, reduced work productivity, and overall compromised health-related quality of life (QOL)^6^. Studies have demonstrated the adverse impact of OIC on health-related QOL in patients with chronic non-cancer pain^6,9^. Additionally, the onset of OIC often complicates opioid therapy, necessitating dose adjustments or complete discontinuation of opioids to alleviate OIC symptoms, thereby further exacerbating chronic pain^10^. Notably, constipation imposes a significant psychosocial burden on patients using opioids. Consequently, further research on the effects of OIC in patients using opioids is imperative, as its significance should not be underestimated^10^.

Gastrointestinal adverse drug reactions, including OIC, have been well-documented with strong opioids. The Rome IV diagnostic criteria for OIC were established for the first time to assess patients with OIC in clinical practice^8,11^. The previous reports have indicated the incidence of OIC based on the Rome IV diagnostic criteria^5,10^. However, these reports primarily aggregated the data for strong opioids, making it difficult to determine the specific incidence for weak opioids. Additionally, studies specifically focusing on weak opioids used for chronic non-cancer pain are limited in Japan. Hence, we hypothesize that a study evaluating OIC incidences per Rome IV diagnostic criteria in patients initiating such treatment would provide real-world insight. This study aimed to clarify the incidence of OIC, diagnosed using the Rome IV diagnostic criteria, in non-cancer patients newly prescribed weak opioids for chronic musculoskeletal pain in routine clinical practice in Japan.

## Methods

### Study design and settings

This observational study was conducted from February 1, 2023, to July 13, 2024, in Japan (trial registration number: UMIN000050203). The study aimed to clarify the incidence of OIC after initiation of weak opioids. This study targeted non-cancer patients who were newly prescribed weak opioids for chronic pain. A web-based, patient-reported questionnaire survey method was used to facilitate participant reporting of OIC. Patients were recruited from 10 orthopedic clinics and 7 orthopedic departments of private, mid-sized hospitals (Supplementary Appendix 1). Prospective participants were provided with a written information sheet of the study at clinics and hospitals and asked to voluntarily submit an online informed consent form via QR codes or a URL outside clinics and hospitals. Upon agreement to participate in the study, participants’ eligibility was confirmed through a web-based survey using the subject enrollment questionnaire (Supplementary Appendix 2). Eligible participants were then requested to complete a self-administered online questionnaire by responding to daily emails. The treating physicians were not informed of the participation of patients in this survey. Laxatives were prescribed according to routine clinical practice. The web-based questionnaire survey was conducted by UNLOG K.K. (Tokyo). To ensure data reliability, this study utilized unique emails for participant tracking.

### Data collection

Deidentified data from the participants, including responses on their demographic and clinical characteristics, as well as baseline data for the Numerical Rating Scale (NRS)^12^ score of pain and the Patient Assessment of Constipation-Symptoms (PAC-SYM)^13^, the validated patient-reported outcome measures, were collected on the enrollment day. Constipation symptoms meeting the Rome IV diagnostic criteria^8^, self-awareness of constipation, medication use, adherence to weak opioid intake, and NRS scores for pain were collected from the start date of initiating weak opioids (day 1) until the last observation day (day 14) (through daily questionnaire; Supplementary Appendix 3). PAC-SYM data were collected at baseline and after 2 weeks (through daily questionnaire; Supplementary Appendixs 3; and weekly surveys assessing bowel movement/symptoms per Rome IV Criteria; Supplementary Appendix 4).

### Study population

The study population consisted of participants aged 18 years and older who were newly prescribed weak opioids for a minimum of 2 weeks; had initiated weak opioids for chronic musculoskeletal pain at the time of enrollment or within the previous day; had experienced at least 3 bowel movements per week before the enrollment day; and were able to complete the online questionnaire using mobile communication devices, such as smartphones and tablets.

Patients were excluded if they were hospitalized; had malignant tumors on the enrollment day; had received opioid treatment within the 4 weeks before enrollment; had fewer than 3 bowel movements per week; or met the Rome IV diagnostic criteria for functional constipation within the past week before enrollment.

### Study endpoints

The primary endpoint was the incidence of OIC during the first 2 weeks. Secondary endpoints included the proportion of participants who experienced each constipation symptom during the first 2 weeks; the responder rate for PAC-SYM; changes from baseline in the total score and scores for each of the 3 domain types (abdominal symptoms, rectal symptoms, and bowel symptoms) for PAC-SYM at the end of the second week; changes from baseline in the NRS score of pain at the end of the second week; and the proportion of participants taking laxatives, as well as the amount of weak opioids taken by participants, including the average number of days on weak opioids and the adherence rate—defined as proportion of days during which weak opioids were taken correctly as prescribed, relative to the total duration of administration—during each observation week. To identify potential predictors of OIC, potential risk factors for the development of OIC were selected based on a previous study^1^ with expert clinical opinion and evaluated as exploratory endpoints, including demographics, physical activity, location of pain, and opioid usage and dosage.

### Assessments

The incidence of OIC was evaluated as the proportion of participants who developed OIC according to the Rome IV diagnostic criteria, with at least 2 of the following constipation symptoms: more than 25% of defecations accompanied by straining, lumpy or hard stools (Bristol Stool Form Scale 1–2), sensation of incomplete evacuation, sensation of anorectal obstruction/blockage, manual maneuvers to facilitate defecation, and/or fewer than 3 bowel movements per week, as recorded in a daily bowel diary. The proportion of participants who experienced each above-mentioned constipation symptom of the Rome IV diagnostic criteria was assessed. The responder rate for PAC-SYM was assessed as the proportion of participants whose total PAC-SYM score increased by ≥ 1 from baseline.

### Sample size

The target sample size was determined based on a feasibility perspective. It was assumed that the number of participants newly prescribed weak opioids for ≥ 2 weeks (referred per doctor) would be 1 per month, based on the information obtained through prior interviews. The calculated achievable sample size of 60 participants was deemed appropriate for this study. No prior data exist on the incidence of OIC using the Rome IV diagnostic criteria in participants newly prescribed weak opioids. As a reference, based on the incidence of constipation as an adverse drug reaction in clinical trials of the tramadol hydrochloride/acetaminophen combination, the incidence of OIC was estimated at 30%. Considering 20% of patients discontinued the use of weak opioids and were never assessed for OIC, it was expected that 14 participants with OIC would be identified.

### Statistical analysis

Participants who met the eligibility criteria for the study and provided responses to the web survey at study start, each follow-up observation day, or the final follow-up observation were included in the analysis. The analysis dataset was then defined by excluding participants who used laxatives prophylactically. Data were analyzed using descriptive statistics; categorical variables were presented as frequency (n) and proportion (%), and continuous variables as mean and standard deviation (SD). For the primary endpoint, the incidence of OIC was expressed as the proportion of participants with OIC within the dataset. OIC onset was determined using the daily bowel movement records for the past 7 days starting from the 7th day after the initiation of weak opioids: if the Rome IV diagnostic criteria for constipation were met, that day was recorded as the day of OIC onset. However, if there were fewer than 4 days with bowel movement records (i.e., days with responses to the daily questionnaire), it was evaluated as “no judgment (missing).” The onset of OIC was considered when 2 or more constipation symptoms, as defined by the Rome IV diagnostic criteria, were present.

Kaplan–Meier (KM) curves, along with 95% confidence intervals (CIs), were used to illustrate the cumulative incidence of OIC, as well as the proportion of participants experiencing each constipation symptom, self-awareness of constipation, and taking laxatives on each day during the first 14 days after initiating weak opioids. For participants who did not develop OIC, experience any constipation symptoms, report self-awareness of constipation, or take laxatives, the censoring date was defined as the last day of the observation period. The proportion of participants taking laxatives during each week (days 1–7 and 8–14) after initiating weak opioids was evaluated, with 95% CIs calculated using the Clopper–Pearson method. In this analysis, the number at risk during each period served as the denominator, and the number of participants who took laxatives served as the numerator.

Summary statistics were calculated for the PAC-SYM and NRS scores at baseline (day 1) and for each week (days 1–7 and 8–14). Changes in scores from baseline to day 14 after the initiation of weak opioids were computed, and comparisons of scores before and after the intervention were made using paired t-tests. Summary statistics for the number of days of weak opioid analgesic use, adherence rates, and their weekly changes (days 1–7 and 8–14) were also calculated.

Furthermore, frequencies and proportions were calculated for qualitative data in the OIC and non-OIC subject groups separately and compared using Fisher’s exact test, unpaired t-test, or the Mann-Whitney U test. The effects of differences in demographic characteristics, medication use, and baseline OIC on the study endpoints were investigated. Univariate logistic regression analysis was conducted to evaluate potential risk factors associated with OIC, using covariates such as sex, age (continuous variable), physical activity, location of pain, and tramadol-equivalent dosage.

In this study, we present the results of 2-sided *p*-values. These *p*-values are not intended for hypothesis testing. Instead, they are provided to illustrate the extent of distributional biases in the frequencies or values between the compared groups or pre- and post-comparisons. Statistical analyses were performed using SAS version 9.4 (SAS Institute, Cary, NC).

Statistical analyses were conducted by an independent statistician who was not involved in the study design or data collection.

### Ethical considerations

The study was conducted in accordance with the ethical principles based on the Declaration of Helsinki and in compliance with the “Ethical Guidelines for Life Science and Medical Research Involving Human Subjects” and the “Guidance,” as well as applicable Japanese laws and regulations. The study was approved by the Takahashi Clinic Ethics Committee of Takahashi Clinic Medical Corporation (trial registration number: UMIN000050203). As this was a web-based survey, the collection of any clinical specimens was not applicable. The purpose of the survey was explained on the web, and electronic consent to participate in this web-based survey was obtained from participants before study commencement.

## Results

### Patient disposition, baseline demographics, and clinical characteristics

**Fig. 1:**
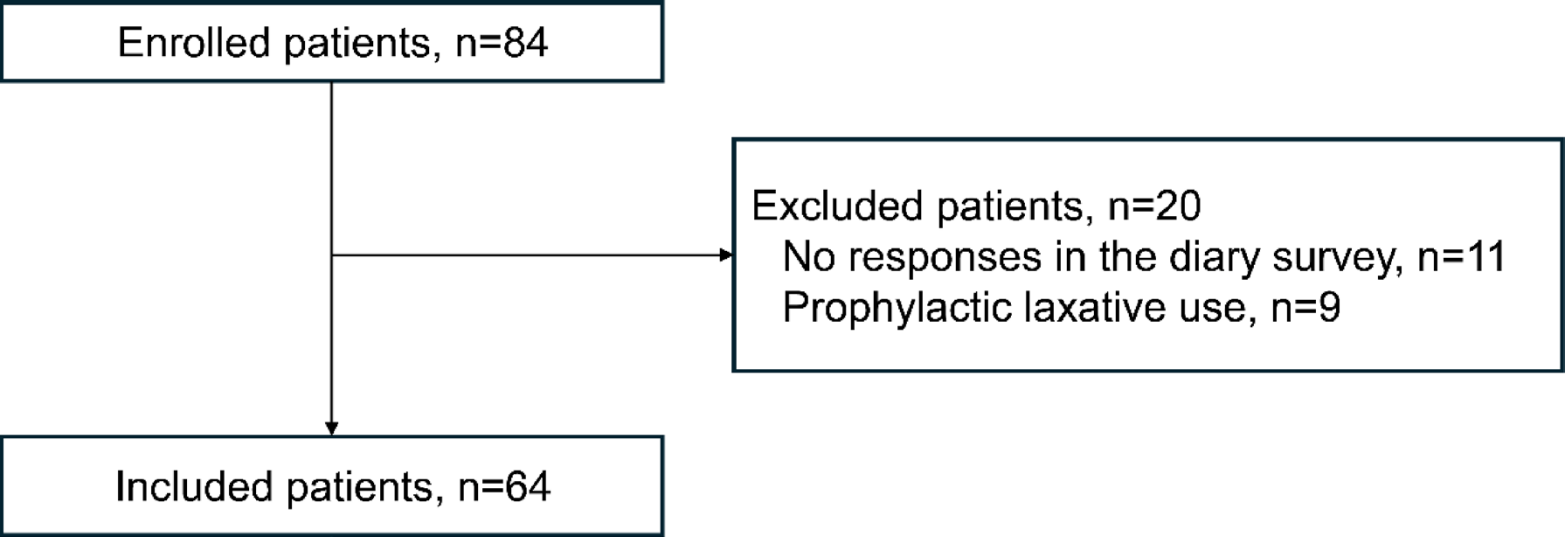
Patient disposition.

Overall, 84 participants were enrolled from February 1, 2023, to July 13, 2024. Of these, 64 participants met the eligibility criteria for the study, while 20 were excluded due to lack of responses in the diary survey or prophylactic laxative use (Fig. 1).

The mean ± SD age of the participants was 53.2 ± 11.3 years, with over 50% being female. Overall, 80% of the participants reported engaging in physical activity for > 1 h/day. A high proportion of participants experienced back pain and lower back pain (35.9%), followed by pain in the lower limbs (29.7%), upper limbs (23.4%), neck (9.4%), and other areas (1.6%). Most participants (64.1%) were prescribed tramadol hydrochloride extended-release (twice a day), followed by tramadol hydrochloride immediate-release (23.4%) and the tramadol hydrochloride/acetaminophen combination (12.5%; Table 1).

**Table 1:** Participant demographics and clinical characteristics.

Characteristic	Participants (*n* = 64)
Age (years), mean ± SD	53.2 ± 11.3
Sex, *n* (%)	
Male	30 (46.9)
Female	34 (53.1)
Physical activity equivalent to or exceeding 1 h of walking per day, *n* (%)	
Yes	51 (79.7)
None	13 (20.3)
Location of pain, *n* (%)	
Back and lower back	23 (35.9)
Lower limbs	19 (29.7)
Upper limbs	15 (23.4)
Neck	6 (9.4)
Other areas	1 (1.6)
Opioid, n (%)	
Tramadol hydrochloride extended-release (twice a day)	41 (64.1)
Tramadol hydrochloride immediate-release	15 (23.4)
Tramadol hydrochloride/acetaminophen combination	8 (12.5)
Weak opioid regimen followed by patients until 2 weeks	
Number of days of weak opioid intake (days/week), mean ± SD	
First week (days 1–7), *n* = 64	6.5 ± 1.1
Second week (days 8–14), *n* = 63	6.6 ± 1.2
Tramadol-equivalent dose (mg/day), mean ± SD	
First week (days 1–7), *n* = 64	67.0 ± 33.9
Second week (days 8–14), *n* = 63	83.9 ± 60.1
Medication adherence rate for weak opioid use (%), mean ± SD	
First week (days 1–7), *n* = 64	98.2 ± 9.0
Second week (days 8–14), *n* = 63	98.2 ± 12.7

SD, standard deviation.

Overall, the adherence rate (mean ± SD) to weak opioids was 98.2 ± 9.0% at 1 week and 98.2 ± 12.7% at 2 weeks. Participants took weak opioids daily as prescribed, with a mean ± SD number of days per week of 6.5 ± 1.1 days in the first week and 6.6 ± 1.2 days in the second week. The mean ± SD tramadol equivalent dose was 67.0 ± 33.9 mg/day in the first week and 83.9 ± 60.1 mg/day in the second week (Table 1).

### Incidence of OIC and OIC symptoms

**Fig. 2:**
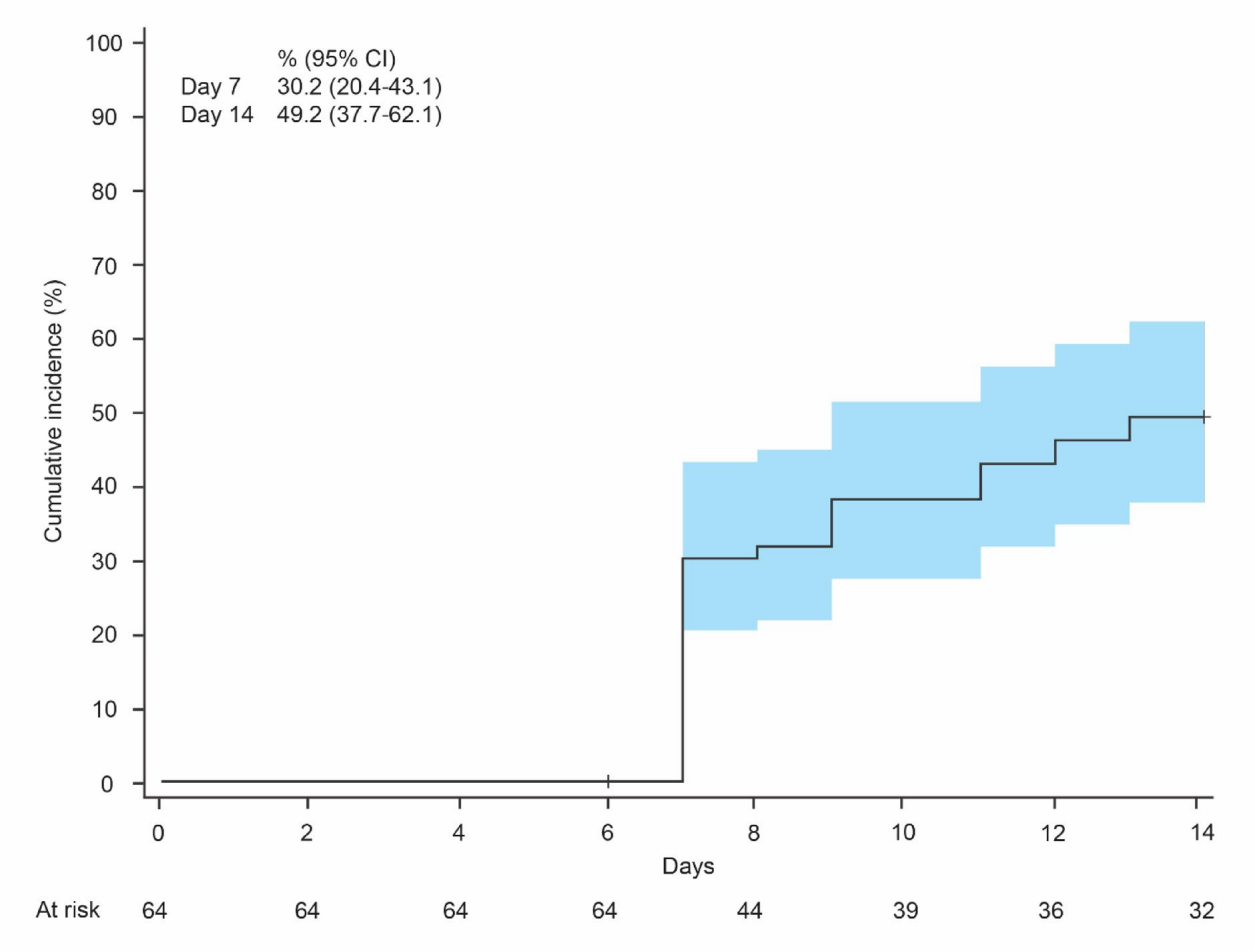
Kaplan–Meier curve for OIC incidence. CI, confidence interval; OIC, opioid-induced constipation. The OIC onset was determined using the daily bowel movement records for the past 7 days starting from the 7th day after the initiation of weak opioids.

The KM curve for the incidence of OIC is shown in Fig. 2. The cumulative incidence of OIC (95% CI) in participants was 30.2% (20.4–43.1) at day 7 and 49.2% (37.7–62.1) at day 14. The prevalence of participants with OIC (95% CI) at day 14 was 32.3% (20.9–45.3; Supplementary Table 1).

**Fig. 3:**
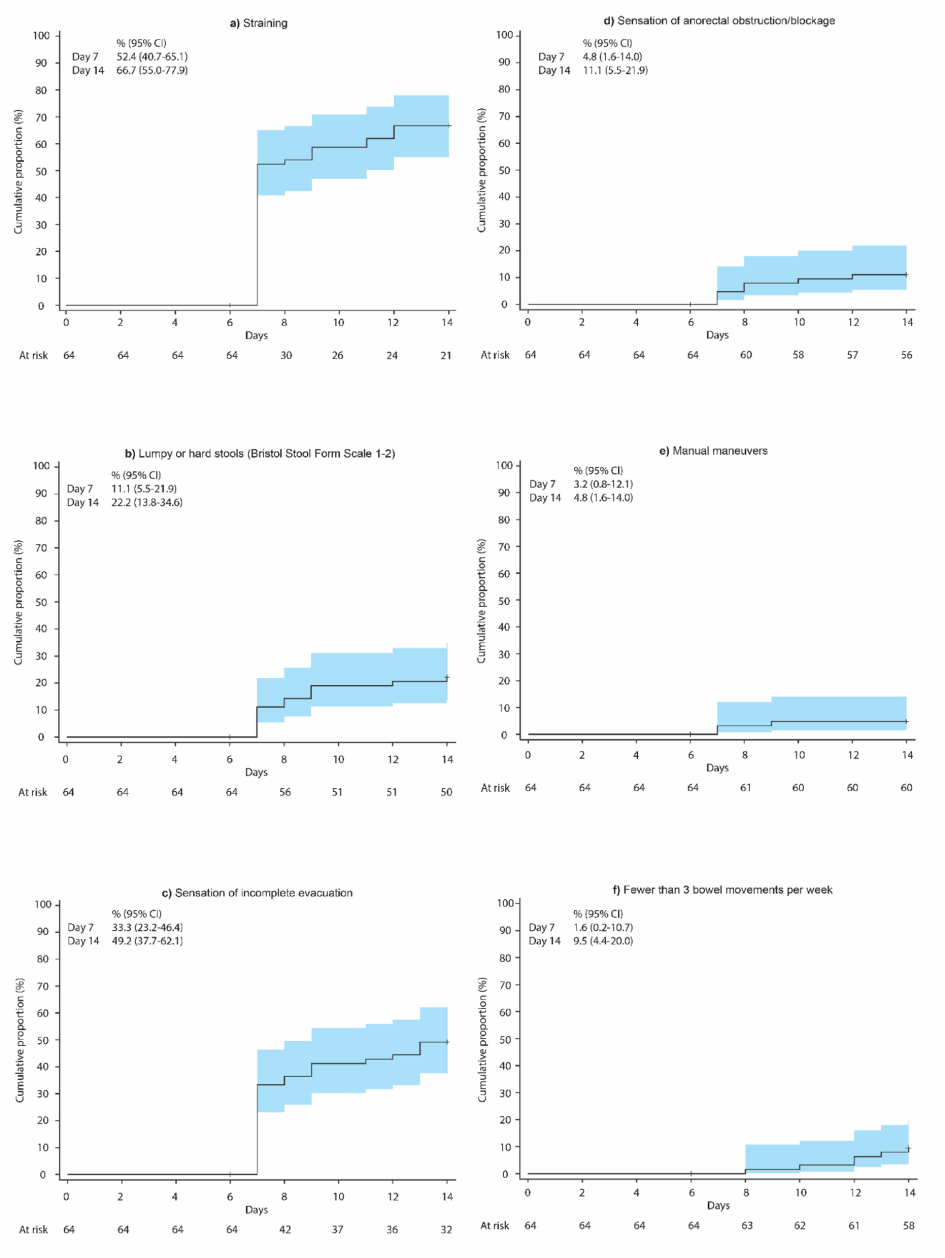
Kaplan–Meier curve for the incidence of OIC symptoms from day 1 until day 14: (**a**) straining, (**b**) lumpy or hard stools (Bristol Stool Form Scale 1–2), (**c**) sensation of incomplete evacuation, (**d**) sensation of anorectal obstruction/blockage, (**e**) manual maneuvers, and (**f**) fewer than 3 bowel movements per week. CI, confidence interval; OIC, opioid-induced constipation. The onset of OIC symptoms was determined using the daily bowel movement records for the past 7 days starting from the 7th day after the initiation of weak opioids.

The KM curves for each constipation symptom are presented in Fig. 3. The cumulative proportion (95% CI) of participants who experienced straining was 52.4% (40.7–65.1) at day 7 and 66.7% (55.0-77.9) at day 14 of initiation of weak opioids, which was higher compared with other constipation symptoms. The cumulative proportion (95% CI) of participants who had sensation of incomplete evacuation was 33.3% (23.2–46.4) at day 7 and 49.2% (37.7–62.1) at day 14, while those who experienced lumpy or hard stools was 11.1% (5.5–21.9) at day 7 and 22.2% (13.8–34.6) at day 14. The cumulative proportion (95% CI) of participants who had sensation of anorectal obstruction was 4.8% (1.6–14.0) at day 7 and 11.1% (5.5–21.9) at day 14, while those who had manual maneuvers and fewer than 3 bowel movements/week were lower at 3.2% (0.8–12.1) and 1.6% (0.2–10.7) at day 7, respectively, and 4.8% (1.6–14.0) and 9.5% (4.4–20.0) at day 14, respectively.

### PAC-SYM scores

PAC-SYM is a valid and responsive measure of the presence and severity of symptoms of OIC. There was a significant increase (worsening) in the total PAC-SYM score (mean ± SD) from day 1 to day 14 (0.28 ± 0.41; *p* < 0.0001), including scores for abdominal symptoms (0.19 ± 0.57; *p* = 0.0116) and defecation symptoms (0.48 ± 0.58; *p* < 0.0001). The responder rate (95% CI) at day 14 after initiation of weak opioids was 8.3% (2.8–18.4; Table 2).

**Table 2:** PAC-SYM scores and responder rate.

	Baseline (day 1) (*n* = 62)^a^	Day 14 (*n* = 62)^a^	Change from baseline (*n* = 60)^b^	*p*-value^c^
PAC-SYM scores, mean ± SD				
Total	0.22 ± 0.28	0.49 ± 0.40	0.28 ± 0.41	< 0.0001
Abdominal symptoms	0.31 ± 0.46	0.50 ± 0.53	0.19 ± 0.57	0.0116
Rectal symptoms	0.11 ± 0.22	0.17 ± 0.32	0.07 ± 0.33	0.0963
Defecation symptoms	0.20 ± 0.32	0.67 ± 0.57	0.48 ± 0.58	< 0.0001
Responders^d^ rate (95% CI), *n*/*N*^b^	8.3 (2.8–18.4), 5/60			

CI, confidence interval; PAC-SYM, Patient Assessment of Constipation-Symptoms; SD, standard deviation.

^a^Participants who answered at baseline or on day 14.

^b^Participants who answered both at baseline and on day 14.

^c^Paired t-test (vs. day 1).

^d^Participants whose PAC-SYM total score increased by ≥ 1 from baseline.

### Self-awareness of constipation

**Fig. 4:**
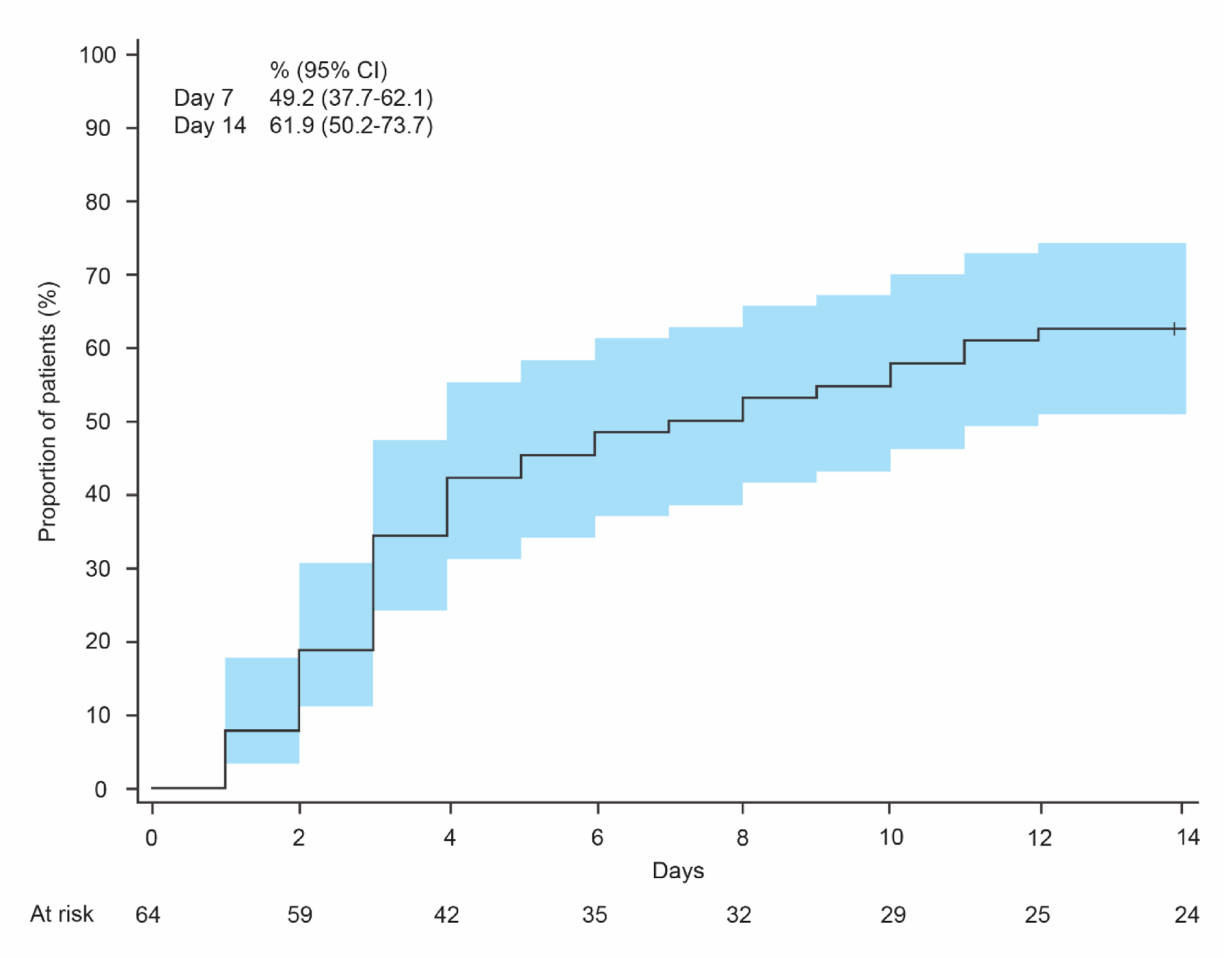
Kaplan–Meier curve for participants with self-awareness of constipation. CI, confidence interval.

The KM curve for the cumulative proportion (95% CI) of participants with self-awareness of constipation increased from 49.2% (37.7–62.1) at day 7 to 61.9% (50.2–73.7) at day 14 (Fig. 4).

### Laxative use in participants

The KM curve for laxative use over 2 weeks showed a gradual increase from day 2 to day 14, with the proportion of participants using laxatives (95% CI) being 31.3% (21.4–44.1) at day 7 and 39.1% (28.3–52.1) at day 14 (Supplementary Fig. 1). Among the types of laxatives used, senna/sennoside (20.3%) use and naldemedine (20.3%) use over 2 weeks were the most common (Supplementary Table 2).

### Association between OIC onset and participant characteristics

The association between the incidence of OIC and patient characteristics is presented in Table 3. Participants with OIC showed similar demographics and clinical characteristics, such as age, sex, physical activity, and location of pain, compared to those without OIC (all *p* > 0.05; Table 3). There was no significant difference in the number of days of weak opioid use, types of weak opioids used, or tramadol-equivalent dosages received between participants with vs. without OIC (all *p* > 0.05; Table 3 and Supplementary Table 3). Baseline pain levels and the analgesic effect of weak opioids (change in NRS) were similar between participants with and without OIC (all *p* > 0.05; Supplementary Table 4).

**Table 3:** Association between the onset of OIC and patient baseline characteristics.

Characteristic	Total (*n* = 63)	OIC (*n* = 31)	Non-OIC (*n* = 32)	*p*-value
Age, *n* (%)				
< 65 years	56 (88.9)	28 (90.3)	28 (87.5)	1.0000^a^
≥ 65 years	7 (11.1)	3 (9.7)	4 (12.5)	
Sex, *n* (%)				
Male	30 (47.6)	14 (45.2)	16 (50.0)	0.8025^a^
Female	33 (52.4)	17 (54.8)	16 (50.0)	
Physical activity, *n* (%)				
Yes	50 (79.4)	24 (77.4)	26 (81.3)	0.7633^a^
None	13 (20.6)	7 (22.6)	6 (18.8)	
Location of pain, *n* (%)				
Back and lower back	23 (36.5)	11 (35.5)	12 (37.5)	1.0000^a^
Lower limbs	19 (30.2)	9 (29.0)	10 (31.3)	1.0000^a^
Upper limbs	14 (22.2)	9 (29.0)	5 (15.6)	0.2374^a^
Neck	6 (9.5)	2 (6.5)	4 (12.5)	0.6719^a^
Other areas	1 (1.6)	0 (0.0)	1 (3.1)	1.0000^a^
Weak opioid usage, mean ± SD				
Number of days taken (days/week)	6.6 ± 1.1	6.4 ± 1.4	6.8 ± 0.6	0.1896^b^
Weak opioids^c^, *n* (%)				
Tramadol hydrochloride extended-release (twice a day)	39 (61.9)	20 (64.5)	19 (59.4)	0.7966^a^
Tramadol hydrochloride immediate-release	16 (25.4)	6 (19.4)	10 (31.3)	0.3870^a^
Tramadol hydrochloride/acetaminophen combination	8 (12.7)	4 (12.9)	4 (12.5)	1.0000^a^
Tramadol-equivalent dose (mg/day), mean ± SD	67.2 ± 34.1	67.7 ± 43.1	66.8 ± 23.0	0.9116^b^
Tramadol-equivalent dose, *n* (%)				
0–100 mg/day	59 (93.7)	28 (90.3)	31 (96.9)	n.t.
101–150 mg/day	2 (3.2)	1 (3.2)	1 (3.1)	
151–200 mg/day	2 (3.2)	2 (6.5)	0 (0.0)	

n.t., not tested; OIC, opioid-induced constipation; SD, standard deviation.

^a^Fisher’s exact test.

^b^Unpaired t-test.

^c^Multiple answers possible/It is possible that the same participants may have reported multiple categories.

### Risk factors for the development of OIC

In the univariate logistic regression analysis, patient characteristics were not identified as significant risk factors for the development of OIC (all *p* > 0.05; Table 4). The results suggest that OIC can occur in participants regardless of sex (crude odds ratio [OR]: 1.21; 95% CI: 0.45–3.27), age (0.96; 0.91–1.01), physical activity (0.79; 0.23–2.69), disease location (back or lower back; 0.92; 0.33–2.56), lower limbs (0.90; 0.31–2.64), upper limbs (2.21; 0.65–7.56), neck (0.48; 0.08–2.85), or weak opioid dosage (1.00; 0.99–1.02; Table 4).

**Table 4:** Association of risk factors for OIC vs. non-OIC in the development of OIC: logistic regression analysis.

	OIC, *n* (*n* = 31)	Non-OIC, *n* (*n* = 32)	Crude OR (95% CI)	*p*-value
Sex^a^				
Male	14	16	1.00 (reference)	
Female	17	16	1.21 (0.45–3.27)	0.7008
Age^a^ (per 1-year-old)	31	32	0.96 (0.91–1.01)	0.0868
Physical activity^a^				
None	7	6	1.00 (reference)	
Yes	24	26	0.79 (0.23–2.69)	0.7075
Location of pain^a^				
Back and lower back (vs. other site)	11	12	0.92 (0.33–2.56)	0.8680
Lower limbs (vs. other site)	9	10	0.90 (0.31–2.64)	0.8480
Upper limbs (vs. other site)	9	5	2.21 (0.65–7.56)	0.2065
Neck (vs. other site)	2	4	0.48 (0.08–2.85)	0.4213
Tramadol-equivalent dose^a^ (mg/day)	31	32	1.00 (0.99–1.02)	0.9089
Tramadol-equivalent dose				
100 mg/day or less	28	31	1.00 (reference)	
More than 100 mg/day	3	1	3.32 (0.33–33.80)	0.3106

CI, confidence interval; OIC, opioid-induced constipation; OR, odds ratio.

^a^Logistic regression analysis: 6 logistic models were applied for each independent variable (sex, age [continuous variable], disease site, and tramadol-equivalent dose [continuous and categorical variable]) and dependent variable (presence or absence of OIC within 2 weeks after initiation of weak opioids) (Yes = 1, No = 0).

## Discussion

This is the first study elucidating the incidence of OIC, based on the Rome IV diagnostic criteria for constipation, in Japanese patients with chronic non-cancer musculoskeletal pain who were newly prescribed weak opioids. The findings revealed that OIC began within a week and increased to nearly 50% within 2 weeks of initiating weak opioids. The most commonly reported OIC symptoms were straining, a sense of incomplete evacuation, and lumpy or hard stools accompanied by fewer bowel movements. Significant changes in PAC-SYM total scores were found for abdominal and defecation symptoms at day 14, with a 8.3% responder rate (total score increased by ≥ 1 from baseline) during the 2 weeks of weak opioid intake. Moreover, the proportion of participants with self-awareness of OIC was 61.9% at day 14. Laxative use was reported by 31.3% of participants by the first week, increasing to 39.1% by the second week of initiating weak opioids. Furthermore, patient baseline and clinical characteristics, as well as weak opioid dosage, were not identified as significant risk factors for the development of OIC in participants with chronic non-cancer pain initiating weak opioids.

The findings of this study are comparable to those reported in patients with cancer who experienced OIC (56% per the Rome IV diagnostic criteria) after initiating strong opioids, thereby confirming that there is no difference in the incidence of OIC between weak and strong opioids^5,14^. Moreover, the significant deterioration observed in PAC-SYM scores, as well as a 8.3% responder rate, objectively demonstrates that the severity of OIC is worsening. The findings of this study reveal that patients had self-awareness of constipation before fulfilling the OIC diagnostic criteria. This also implies that when prescribing weak opioids, physicians can identify OIC by assessing patient awareness.

In the present study, the opioid adherence rate was approximately 100%, and the amount of weak opioids taken was lower (average dose of tramadol equivalent: 67.2 mg/day) than the approved minimum daily dose of 100 mg/day of tramadol in Japan. This dosage is consistent with a previous study, which reported that the average dose of tramadol is 50 mg/day in patients with osteoarthritis and 67 mg/day in patients with chronic lower back pain in Japan^15^. The Japanese guidelines recommend considering doses lower than 100 mg/day to avoid side effects, which may be influencing this^2^. Notably, the present study showed that the dose of weak opioids did not influence the development of OIC. These findings highlight the need for awareness of OIC, regardless of the prescribed dosage of weak opioids, to help prevent negative perceptions of opioid therapy and improve chronic pain management^1^.

This study also revealed that OIC symptoms such as straining, a sense of incomplete evacuation, and hard stools were more common than reduced bowel movements experienced by participants. These findings align with previous reports in healthy participants^16^ and in chronic non-cancer patients^1^. Thus, it is important to assess patients for symptoms such as straining, a sense of incomplete evacuation, and hard stools, in addition to the number of bowel movements^1,16^. Opioids may contribute to symptoms such as straining and a sense of incomplete evacuation due to their inhibition of intestinal peristalsis and effects on anal sphincter tension^16,17^. It has also been reported that tramadol use delays colonic transit time, reduces daily bowel movements, and results in ineffective evacuations and hard stools^16^.

In a previous study of patients with chronic non-cancer pain, 30% of patients reported self-assessed constipation after initiating opioids^1^. However, that study was a single-point survey, without information on the initiation of opioid treatment. Additionally, some patients were using laxatives, and others had constipation before opioid intake^1^. In contrast, the present study identified the onset of OIC with the intake of weak opioids by only including participants newly prescribed weak opioids and excluding those who experienced constipation before weak opioid use.

In the present study, laxatives were taken by 31.3% of participants during the first week and 39.1% by the end of the second week of initiating weak opioids. Participants with prophylactic laxative use were excluded, implying that the laxatives reported in the study were taken for the treatment of OIC. The cumulative incidence of OIC continued to increase in a time-dependent manner from day 7 to day 14, without reaching a plateau. However, the proportion of participants with OIC at each time point did not increase after day 7, suggesting that some cases of OIC may have resolved with treatment. Senna/sennosides and naldemedine were the most commonly prescribed treatments for participants with OIC. The Japanese guidelines recommend naldemedine if conventional laxatives (osmotic or stimulant) are inadequate in managing OIC^18^. The role of peripheral µ-opioid receptors in tramadol-induced constipation is well-documented in a preclinical study involving rodents^19^. Consequently, the administration of a peripherally acting µ-opioid receptor antagonist, such as naldemedine, presents an effective approach for selectively and locally antagonizing the gastrointestinal effects of opioids without compromising systemic analgesia^20,21^. In addition, there was no difference in pain reduction after initiating weak opioids between participants with OIC and those without OIC. This suggests that participants with OIC in this study received appropriate treatment for OIC, which did not affect the analgesic effect of weak opioids.

In this study, no differences were identified in patient characteristics between participants with OIC and those without OIC. The development of OIC was not associated with age, sex, physical activity, location of pain, or weak opioid dosage. However, in a previous study of Japanese patients with chronic non-cancer pain, lower back pain was identified as a risk factor (OR: 3.17) for developing OIC^1^, but that study included patients who had been constipated before taking opioids. A cross-sectional survey on women’s health in Australia reported that multiple gastrointestinal symptoms were significantly associated with back pain in women^22^, suggesting that patients with lower back pain are predisposed to constipation and are more likely to develop OIC after initiating opioid treatment^1,22^.

The study has some limitations, which are primarily intrinsic to its design. The study was conducted using a web-based survey, with results based solely on patient-reported outcomes. There has been no constipation diagnosis by a physician. Additionally, participation was limited to relatively young patients who could use smartphones, tablets, or other devices, as responses were collected online through an application. All participants are taking weak opioids, and there was no control group. The questions used in this study were based on the Rome IV diagnostic criteria, but they have been modified for a web survey and are not a validated questionnaire. The small sample size inhibited a comprehensive analysis of risk factors. Furthermore, the Rome IV diagnostic criteria used in this study require a 7-day observation period to diagnose OIC; therefore, it is not possible to make a temporal diagnosis of OIC until the 7th day from the start of observation. However, if laxatives are taken before meeting the diagnostic criteria for OIC, there is a possibility that the onset of OIC could be prevented. Furthermore, participants were informed in advance about the potential development of OIC, which may increase the proportion of participants experiencing OIC or decrease it due to modifications in their dietary habits, such as increasing their dietary fiber intake.

However, since the survey was conducted daily and evaluated changes from the time of registration, it is believed that participants’ understanding of the questions did not significantly affect the results. In addition, the potential for treatment bias was minimized in this study, as the treating physicians were not informed of or aware about the participation of participants in this survey.

Despite these limitations, by recording daily bowel movement status, this study elucidated the onset of OIC and the emergence of each symptom for the first time in Japanese patients with chronic non-cancer pain. It also underscored the importance of assessing not only the frequency of bowel movements but also each symptom of constipation, regardless of the prescribed opioid dosage.

## Conclusion

The study emphasized that a high rate of OIC may occur even with short-term use of a small dose of weak opioids, highlighting the importance of monitoring OIC when initiating pain management using weak opioids. The study also revealed the emergence of constipation symptoms and the need for OIC awareness in patients with chronic non-cancer pain in Japan.

## Electronic supplementary material

Below is the link to the electronic supplementary material.

Supplementary Material 1.

## Data Availability

The datasets generated and/or analyzed during the current study are available from the corresponding author upon reasonable request.

## References

[1] Sonohata, M. et al. A survey of the incidence of constipation in patients with chronic non-cancer pain using opioid analgesics in Japan. *Pain Ther.* **11**, 845–859 (2022). 35598289 10.1007/s40122-022-00392-y PMC9314494

[2] Japan Society of Pain Clinicians. Opioid Analgesic Prescribing Guidelines for Non-Cancer Chronic Pain. https://www.jspc.gr.jp/Contents/public/kaiin_guideline04.html. Accessed 30 October 2024.

[3] Sonohata, M., Kitamura, M., Hashimoto, A. & Morioka, Y. Prescription pattern of laxatives for opioid-induced constipation in Japanese patients with chronic non-cancer pain: A retrospective cohort study of a health insurance claims database. *Cureus.* **17**, e78212 (2025). 40027044 10.7759/cureus.78212 PMC11871374

[4] Kumar, L., Barker, C. & Emmanuel, A. Opioid-induced constipation: Pathophysiology, clinical consequences, and management. *Gastroenterol. Res. Pract.* 141737 (2014). 10.1155/2014/141737 PMC4027019 24883055

[5] Tokoro, A. et al. Incidence of opioid-induced constipation in Japanese patients with cancer pain: A prospective observational cohort study. *Cancer Med.* **8**, 4883–4891 (2019). 31231974 10.1002/cam4.2341 PMC6712473

[6] Coyne, K. S. et al. Opioid-induced constipation in patients with chronic non-cancer pain in the USA, Canada, Germany, and the UK: Descriptive analysis of baseline patient-reported outcomes and retrospective chart review. *Clinicoecon Outcomes Res.* **6**, 269–281 (2014). 24904217 10.2147/CEOR.S61602 PMC4041290

[7] Kanemasa, T. et al. Pharmacologic effects of naldemedine, a peripherally acting µ-opioid receptor antagonist, in in vitro and in vivo models of opioid‐induced constipation. *Neurogastroenterol. Motil.* **31**, e13563 (2019). 30821019 10.1111/nmo.13563 PMC6850587

[8] Drossman, D. et al. *The Rome IV Committees. Rome IV Functional Gastrointestinal Disorders – Disorders of Gut-Brain Interaction* (The Rome Foundation, 2016).

[9] Kitamura, M., Morioka, Y., Kobayashi, M. & Ushida, T. A web–based survey on the quality of life of patients with opioid induced constipation using weak opioids in chronic non–cancer musculoskeletal pain in Japan. *PAIN Res.* 64–75 (2024).

[10] Andresen, V., Banerji, V., Hall, G., Lass, A. & Emmanuel, A. V. The patient burden of opioid-induced constipation: New insights from a large, multinational survey in five European countries. *United Eur. Gastroenterol. J.* **6**, 1254–1266 (2018). 10.1177/2050640618786145 PMC6169046 30288288

[11] Schmulson, M. J. & Drossman, D. A. What is new in Rome IV. *J. Neurogastroenterol Motil.* **23**, 151–163 (2017). 28274109 10.5056/jnm16214 PMC5383110

[12] Farrar, J. T., Young, J. P., LaMoreaux, L., Werth, J. L. & Poole, M. R. Clinical importance of changes in chronic pain intensity measured on an 11-point numerical pain rating scale. *Pain.* **94**, 149–158 (2001). 11690728 10.1016/S0304-3959(01)00349-9

[13] Slappendel, R., Simpson, K., Dubois, D. & Keininger, D. L. Validation of the PAC-SYM questionnaire for opioid-induced constipation in patients with chronic low back pain. *Eur. J. Pain.* **10**, 209 (2006). 15914049 10.1016/j.ejpain.2005.03.008

[14] Fumita, S. et al. Patients’ self-assessment of the symptoms and impact of opioid-induced constipation: Results from a prospective observational cohort study of Japanese patients with cancer. *J. Pain Symptom Manag.* **59**, 1043–1051e2 (2020). 10.1016/j.jpainsymman.2019.11.021 31805362

[15] Akazawa, M. et al. Patterns of drug treatment in patients with osteoarthritis and chronic low back pain in Japan: A retrospective database study. *J. Pain Res.* **12**, 1631 (2019). 31190973 10.2147/JPR.S203553 PMC6535438

[16] Larsen, I. M., Okdahl, T., Mark, E. B., Frøkjær, J. B. & Drewes, A. M. The influence of tramadol on bowel function: A randomised, placebo-controlled trial. *Basic. Clin. Pharmacol. Toxicol.* **135**, (2024). 10.1111/bcpt.14067 39168825

[17] Müller-Lissner, S. et al. Opioid-induced constipation and bowel dysfunction: A clinical guideline. *Pain Med.* **18**, 1837–1863 (2017). 28034973 10.1093/pm/pnw255 PMC5914368

[18] Mawatari, H., Shinjo, T., Morita, T., Kohara, H. & Yomiya, K. Revision of pharmacological treatment recommendations for cancer pain: Clinical guidelines from the Japanese society of palliative medicine. *J. Palliat. Med.* **25**, 1095–1114 (2022). 35363057 10.1089/jpm.2021.0438

[19] Yasufuku, K. et al. Involvement of the peripheral µ-opioid receptor in tramadol-induced constipation in rodents. *Biol. Pharm. Bull.* **44**, 1746–1751 (2021). 34719650 10.1248/bpb.b21-00474

[20] Pergolizzi, J. V., Christo, P. J., Lequang, J. A. & Magnusson, P. The use of peripheral µ-opioid receptor antagonists (PAMORA) in the management of opioid-induced constipation: An update on their efficacy and safety. *Drug Des. Devel Ther.* **14**, 1009–1025 (2020). 32210534 10.2147/DDDT.S221278 PMC7075239

[21] Rekatsina, M. et al. Efficacy and safety of peripherally acting µ-opioid receptor antagonist (PAMORAs) for the management of patients with opioid-induced constipation: A systematic review. *Cureus.* **13** (2021). 10.7759/cureus.16201 PMC8339109 34367804

[22] Smith, M. D., Russell, A. & Hodges, P. W. How common is back pain in women with Gastrointestinal problems? *Clin. J. Pain.* **24**, 199–203 (2008). 18287824 10.1097/AJP.0b013e31815d3601

