# Author Correction: Incidence of opioid-induced constipation in non-cancer patients using weak opioids for chronic pain in Japan: a cohort study

**DOI:** 10.1038/s41598-025-24259-2

**Published:** 2025-10-27

**Authors:** Akira Hashimoto, Yasuhide Morioka, Shihomi Wada, Yuichi Koretaka, Motoki Sonohata

**Affiliations:** 1 Department of Orthopaedic Surgery, Japan Community Health Care Organization (JCHO) Saga Central Hospital, 3-8-1, Hyogominami, Saga City, Saga 849-8522 Japan; 2 Medical Affairs Department, Shionogi & Co., Ltd., Nissay Yodoyabashi East, 3-13, Imabashi 3-chome, Chuo-ku, Osaka, 541-0042 Japan; 3 Data Science Department, Shionogi & Co., Ltd., 4F MTR building, 3-6-3, Awajimachi, Chuo-ku, Osaka, 541-0047 Japan

Correction to: *Scientific Reports* 10.1038/s41598-025-01770-0, published online 13 June 2024

The original version of this Article contained errors in Table 2, where due to a miscalculation, the sum of the scores were computed instead of the average.

The correct and incorrect values appear below.

Table 2:

Incorrect:

**Table:** 

	**Baseline (day 1) (*n*=62)**^**a**^	**Day 14 (*n*=62)**^**a**^	**Change from baseline (*n*=60)**^**b**^	***p*-value**^**c**^
PAC-SYM scores, mean±SD				
Total	2.6 ± 3.4	5.9 ± 4.8	3.4 ± 5.0	<0.0001
Abdominal symptoms	1.2 ± 1.9	2.0 ± 2.1	0.8 ± 2.3	0.0116
Rectal symptoms	0.3 ± 0.7	0.5 ± 1.0	0.2 ± 1.0	0.0963
Defecation symptoms	1.0 ± 1.6	3.4 ±2 .8	2.4 ± 2.9	<0.0001
Respondersd rate (95% CI), *n*/*N*b	70.0 (56.8–81.2), 42/60			

Correct:

**Table:** 

	**Baseline (day 1) (*n*=62)**^**a**^	**Day 14 (*n*=62)**^**a**^	**Change from baseline (*n*=60)**^**b**^	***p*-value**^**c**^
PAC-SYM scores, mean±SD				
Total	0.22 ± 0.28	0.49± 0.40	0.28 ± 0.41	<0.0001
Abdominal symptoms	0.31 ± 0.46	0.50 ± 0.53	0.19 ± 0.57	0.0116
Rectal symptoms	0.11 ± 0.22	0.17 ± 0.32	0.07 ± 0.33	0.0963
Defecation symptoms	0.20 ± 0.32	0.67 ± 0.57	0.48 ± 0.58	<0.0001
Respondersd rate (95% CI), *n*/*N*b	8.3 (2.8-18.4), 5/60			

The original Article has been corrected.

