# Supplementary material for: Incidence of opioid-induced constipation in non-cancer patients using weak opioids for chronic pain in Japan: a cohort study
Source: Sci Rep. 2025 May 19;15:17397. doi: 10.1038/s41598-025-01770-0 (PMC12089302; doi:10.1038/s41598-025-01770-0)
Supplement: Supplementary file 1 — Supplementary Material 1. [file 41598_2025_1770_MOESM1_ESM.pdf]

Supplementary information

Type of article: Article

Title: Incidence of opioid-induced constipation in non-cancer patients using weak opioids for chronic pain in Japan: a cohort study.

Authors:

Akira Hashimoto[1], Yasuhide Morioka[2,*], Shihomi Wada[2], Yuichi Koretaka[3], Motoki Sonohata[1]

Author affiliations:

[1]Department of Orthopaedic Surgery, Japan Community Health Care Organization (JCHO) Saga Central Hospital, 3-8-1, Hyogominami, Saga City, Saga 849-8522, Japan

[2]Medical Affairs Department, Shionogi & Co., Ltd., Nissay Yodoyabashi East, 3-13, Imabashi 3-chome, Chuo-ku, Osaka 541-0042, Japan

[3]Data Science Department, Shionogi & Co., Ltd., 4F MTR building, 3-6-3, Awajimachi, Chuo-ku, Osaka 541-0047, Japan

***Corresponding author:**

Yasuhide Morioka

Medical Affairs Department, Shionogi & Co., Ltd.,

Nissay Yodoyabashi East, 3-13, Imabashi 3-chome, Chuo-ku, Osaka 541-0042, Japan

Email address: yasuhide.morioka@shionogi.co.jp

Contact number: +81-70-7812-6306

ORCID iD: 0000-0001-7878-2557

Supplementary appendix 1. Collaborators for patient recruitment

Name	Clinic/hospital
Akira Onda	Department of Orthopaedic Surgery, Zenshukai Hospital, Gunma, Japan
Dai Matsumoto	Dai Orthopaedic Clinic, Sapporo, Hokkaido, Japan
Eijiro Okada	Setagaya Okada Orthopaedic Surgery Clinic, Tokyo, Japan
Kazuhiko Maeda	Maeda Orthopaedics and Internal Medicine, Kanagawa, Japan
Kengo Nishio	Nishio Hospital, Fukuoka, Japan
Koji Kawai	Ishinomaki Branch, Sendai Pain Clinic Center, Higashi Matsushima, Miyagi, Japan
Osamu Kaneko	Kaneko Seikei, Yokohama, Japan
Satoru Nakajo	Nakajo Orthopaedic Clinic, Sendai, Miyagi, Japan
Shinnichi Ri	Noda Hospital, Chiba, Japan
Takashi Kamiyama	Ooedoseikeigekanaika, Tokyo, Japan
Takayoshi Tanaka	Tanaka Orthopaedic Clinic, Yokohama, Japan
Takeshi Shutta	Hospital of Settu Hikari, Osaka, Japan
Terumasa Nakatsuka	Nakatsuka Orthopaedic Clinic, Osaka, Japan
Tsuneo Takebayashi	Sapporo Maruyama Orthopedics Hospital, Hokkaido, Japan
Wataru Taniguchi	Kotonoura Rehabilitation Center, Wakayama, Japan
Yoichiro Matsuo	Matsuo Orthopaedic & Rehabilitation Clinic, Miyoshi, Hiroshima, Japan
Anonymous	Osaka, Japan

Supplementary appendix 2. Patient enrollment questionnaire

NU, numerical answer; SA, single answer.

Eligibility criteria check (selection criteria)

1. Sex (SA)
    a. Male
    b. Female
2. What is your age? (NU)
3. In your daily life, do you walk or engage in equivalent physical activity for at least 1 hour per day? (SA)
    a. Yes
    b. No
4. Please tell us the area of pain* that was targeted by the painkiller you were prescribed this time. (SA)

    *Pain is limited to pain related to joints, muscles, and bones.
    a. Neck pain
    b. Back and lower back pain
    c. Pain in the upper limbs (shoulders, arms, hands)
    d. Pain in the lower limbs (thighs, knees, lower legs, feet)
    e. Pain in other areas
    f. No pain in joints, muscles, or bones
5. Have you taken any of the following painkillers* in the past 4 weeks? (SA)

    *This does not include prescription painkillers.
    a. Yes
    b. No

MS Contin Tablets, Morpes Granules, MS Twaislon Capsules, Paseef Capsules, Kadian Capsules (morphine hydrochloride hydrate)
Abstral sublingual tablets, Ephen buccal tablets (fentanyl citrate)
OxyContin TR Tablets (Oxycodone Extended Release)
Oxycodone Powder
Codeine Phosphate
Tapenta Tablets (Tapentadol Hydrochloride)
Twotram Tablets (Tramadol hydrochloride extended release)

DuroTep MT Patch, One Duro Patch (Fentanyl)
Toaraset Combination Tablets (Tramadol Hydrochloride/Acetaminophen)
Tramal OD Tablets
Tramset combination tablets (tramadol hydrochloride and acetaminophen)
Narsus Tablets (hydromorphone hydrochloride extended release)
Narrapid Tablets (Hydromorphone Hydrochloride)
Norspan Tape (Buprenorphine)
Fentanyl citrate 1-day tape, Fentanyl 1-day tape, Fentanyl 3-day tape (Fentanyl)
Fentanyl Tape (Fentanyl Citrate)
Methapain Tablets (Methapain Hydrochloride)
Morphine hydrochloride tablets, morphine hydrochloride powder (morphine hydrochloride)
Onetram Tablets (Tramadol Hydrochloride Extended Release)

6. What type of weak opioid painkiller was prescribed this time? (SA)
    a. Codeine phosphate
    b. Twotram tablets (tramadol hydrochloride extended-release)
    c. Toaraset combination tablets (tramadol hydrochloride/acetaminophen combination)
    d. Tramal once-a-day tablets (tramadol hydrochloride extended-release)
    e. Tramset combination tablets (tramadol hydrochloride/acetaminophen combination)
    f. Onetram tablets (tramadol hydrochloride extended-release)
    g. Medications other than those listed above
7. Regarding the prescribed medication in the previous question, do you think you would continue taking it for more than 2 weeks if there were no particular problems? (SA)
    a. Yes
    b. No

Eligibility criteria check (exclusion criteria)

8. When did you start or will start taking the weak opioid painkiller prescribed this time? (SA)
    a. Taken today or to be taken today
    b. Yesterday
    c. More than 2 days ago
    d. I will start taking it tomorrow

9. Are you currently hospitalized? (SA)
    a. Yes
    b. No
10. How many times in total have you had a bowel movement in the past week? (SA)
    a. 0, 1, 2, 3, 4, 5, 6, 7 or more pull-downs
11. Are you currently suffering from cancer? (SA)
    a. Yes
    b. No
12. Regarding your bowel movements over the past week, have 2 or more of the following conditions applied to you? (SA)

・ Straining during bowel movements (1 in 4)

・1 of every 4 bowel movements contains rabbit droppings or hard stools (corresponding to 1 or 2 in the table)

・Feeling like there is still stool remaining after each defecation (1 in 4 times)

・Feeling of blockage or congestion in the rectum and anus during bowel movements (1 in 4)

・When defecation occurs (1 in 4 times), the patient uses their fingers to scrape out the bowels or applies pressure to the lower abdomen or perineum

・Fewer than 3 bowel movements per week

    a. Yes
    b. No
13. Please check the appropriate box for your bowel movement status over the past week in the previous question (SA)

    Choices

    a. Straining during bowel movements (1 in 4)
    b. 1 of every 4 bowel movements contains rabbit droppings or hard stools (corresponding to 1 or 2 in the table)
    c. Feeling like there is still stool remaining after 1 in 4 bowel movements
    d. Sensation of rectal blockage or congestion during bowel movements (1 in 4)
    e. When defecation occurs (1 in 4 times), the patient uses their fingers to scrape out the bowels or applies pressure to the lower abdomen or perineum
    f. Fewer than 3 bowel movements per week
    g. None of the above

Supplementary appendix 3. Daily survey

FA, free answer; MA, multiple answers; SA, single answer.

Research subject information (necessary for answer confirmation and reminders)

1. Email address (FA)
    a. Input
2. Name (please enter correctly in Kanji) (FA)
    a. Input

Check bowel movement status

3. Do you feel constipated today? (SA)
    a. Yes
    b. No
4. Did you have a bowel movement today? (SA)
    a. Yes → Go to 5
    b. No → Go to 11 (medication confirmation)

Please tell me about your bowel movements today

5. Please tell me how many bowel movements you had today (SA)
    a. Pull-down from 1 to 10
6. Please enter the number of bowel movements that involved straining today (SA)
    a. Pull-down from 0 to 10
7. Please enter the number of bowel movements today that were accompanied by a feeling of "residual bowel movement" (SA)
    a. Pull-down from 0 to 10
8. Please enter the number of bowel movements today that required you to "scoop out the bowels with your fingers or apply pressure to the lower abdomen or perineum" (SA)
    a. Pull-down from 0 to 10
9. Please enter the number of times during today's bowel movements that you felt a "blockage or congestion in the rectum or anus" (SA)
    a. Pull-down from 0 to 10
10. Please check the most appropriate option for the shape of your flight today (SA)
    a. 1~7

Medication confirmation

Could you please tell me about your medication today?

11. Did you take any laxatives today? (SA)

a. Yes → Go to 12 (only those who have taken laxatives [branch])
b. No → Go to 13

Only those who have taken laxatives (branch)

12. Please tell us what laxative you took today (MA)
    a. Magnesium oxide, magnesium corrol (magnesium oxide)
    b. Movicol (macrogol)
    c. Pulsenide, arosene, yodel (senna, sennoside)
    d. Thereminsoft, picosulphate (bisacodyl, picosulphate)
    e. Amitiza (lubiprostone)
    f. Linzess (linaclotide)
    g. Goofice (elobixibat)
    h. Daikenchuto, daiokanzoto (Chinese herbal medicine)
    i. Symproic (naldemedine)
    j. Other laxatives besides those mentioned above
13. Did you take any of the following weak opioid painkillers today? (SA)

    Codeine phosphate (codeine phosphate)

    Twotram tablets (tramadol hydrochloride extended-release)

    Toaraset combination tablets (tramadol hydrochloride/acetaminophen combination)

    Tramal once-a-day tablets (tramadol hydrochloride extended-release)

    Tramset combination tablets (tramadol hydrochloride/acetaminophen combination)

    Onetram tablets (tramadol hydrochloride extended-release)
    a. Yes → Go to 14 (only those who took painkillers [branch])
    b. Go to 17 pain assessment (Numerical Rating Scale)

Only those who took painkillers (branch)

14. What is the name of the weak opioid painkiller you took today? (MA)
    a. Codeine phosphate (codeine phosphate)
    b. Twotram tablets 50 mg (tramadol hydrochloride extended-release)
    c. Twotram tablets 100 mg (tramadol hydrochloride extended-release)
    d. Twotram tablets 150 mg (tramadol hydrochloride extended-release)
    e. Toaraset combination tablets (tramadol hydrochloride/acetaminophen combination)
    f. Tramal once-a-day tablets 25 mg (tramadol hydrochloride extended-release)
    g. Tramal once-a-day tablets 50 mg (tramadol hydrochloride extended-release)

h. Tramset combination tablets (tramadol hydrochloride/acetaminophen combination)

i. Onetram tablets 100 mg (tramadol hydrochloride extended-release)

15. Did you take your weak opioid painkillers as prescribed? (SA)

 a. Yes

 b. No

16. How many tablets of weak opioid painkillers did you take today? (SA)

 a. Pull-down from 1 to 20 tablets

Pain rating scale (Numerical Rating Scale)

17. Please choose your average condition today, with "0" being no pain and "10" being the worst pain you can imagine (SA)

 ○ (no pain) 0 1 2 3 4 5 6 7 8 9 10 (worst pain)

Supplementary appendix 4. Weekly survey

SA, single answer.

Rome IV (diagnostic criteria for opioid-induced constipation)

Please select all that applies regarding your bowel movements over the past week

1. Straining during bowel movements (1 in 4) (SA)

    a. Yes

    b. No

2. 1 in 4 bowel movements contains rabbit droppings or hard stools (corresponding to 1 or 2 in the table) (SA)

    a. Yes

    b. No

3. Feeling of incomplete bowel movement (1 in 4 times) (SA)

    a. Yes

    b. No

4. Sensation of anorectal blockage or congestion during bowel movements (1 in 4) (SA)

    a. Yes

    b. No

5. When defecation occurs (1 in 4 times), the patient is forced to push the bowel out with their fingers or to apply pressure to the lower abdomen or perineum (SA)

    a. Yes

    b. No

6. Fewer than 3 bowel movements per week (SA)

    a. Yes

    b. No

Supplementary Fig. S1. Kaplan-Meier curve for laxative use

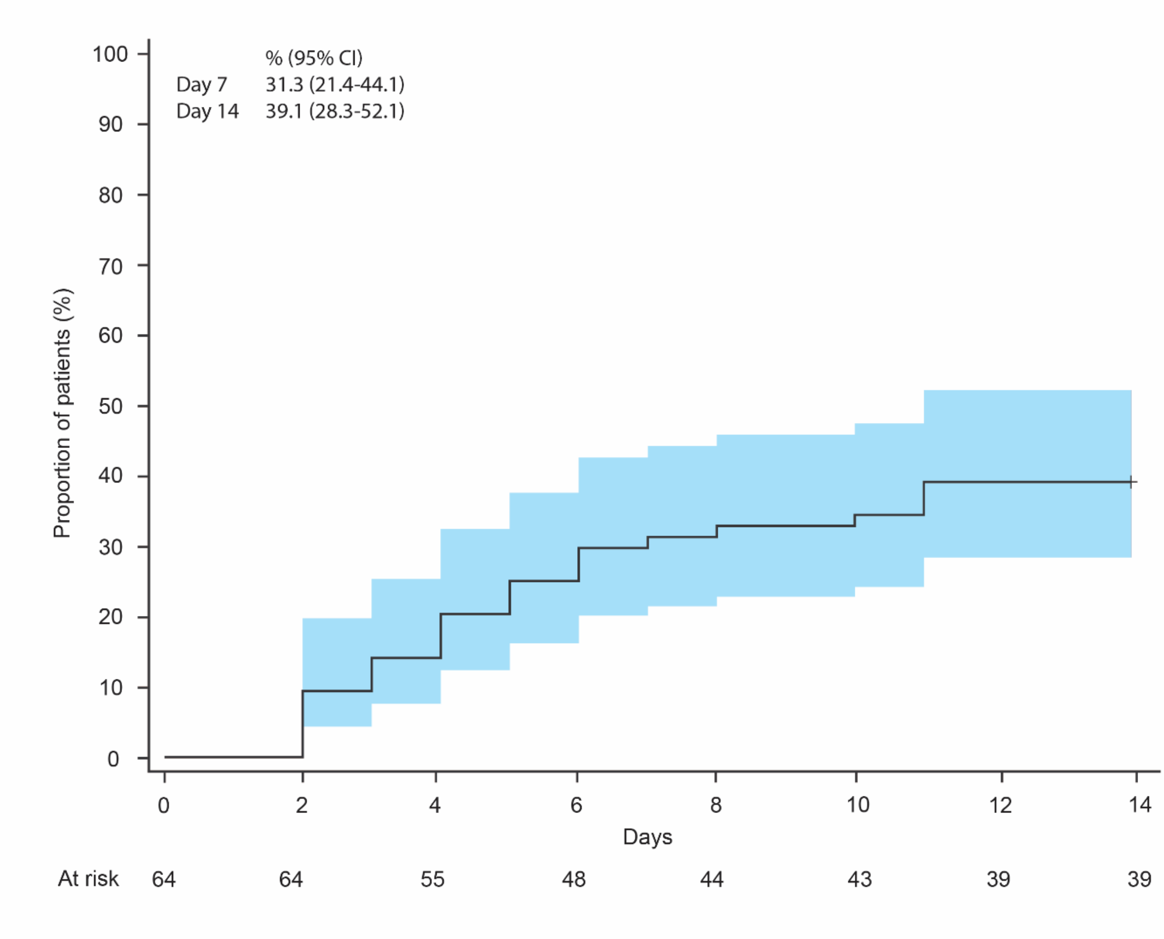

CI, confidence interval.

Supplementary Table 1. Prevalence of OIC on each day

	Total[a]	OIC[b]	Prevalence (95% CI)[c]
Day 7	63	19	30.2 (19.2-43.0)
Day 8	63	19	30.2 (19.2-43.0)
Day 9	63	24	38.1 (26.1-51.2)
Day 10	63	21	33.3 (22.0-46.3)
Day 11	63	21	33.3 (22.0-46.3)
Day 12	62	22	35.5 (23.7-48.7)
Day 13	62	23	37.1 (25.2-50.3)
Day 14	62	20	32.3 (20.9-45.3)

CI, confidence interval; OIC, opioid-induced constipation.

[a]The number of participants who could be assessed for OIC on each day.

[b]The number of participants who met the diagnostic criteria for OIC on each day.

[c](Proportion of OIC participants/total number of participants)×100

Supplementary Table 2. Participants using each laxative

Characteristic	Total (n=64)	First week (days 2-7) (n=64)	Second week (days 8-14) (n=63)
Laxative use, n (%)[a]	25 (39.1)	20 (31.3)	23 (36.5)
Senna/sennoside	13 (20.3)	11 (17.2)	10 (15.9)
Naldemedine	13 (20.3)	8 (12.5)	12 (19.0)
Magnesium oxide	2 (3.1)	1 (1.6)	1 (1.6)
Bisacodyl, sodium picosulphate	1 (1.6)	0 (0.0)	1 (1.6)
Others	1 (1.6)	1 (1.6)	1 (1.6)
Magnesium citrate	0 (0.0)	0 (0.0)	0 (0.0)
Lubiprostone	0 (0.0)	0 (0.0)	0 (0.0)
Linaclotide	0 (0.0)	0 (0.0)	0 (0.0)
Elobixibat	0 (0.0)	0 (0.0)	0 (0.0)
Daikenchuto (Kampo)	0 (0.0)	0 (0.0)	0 (0.0)

[a]The denominator is the number of cases, and the number shown represents participants who took the medication at least once during the specified period.

Supplementary Table 3. Tramadol-equivalent dose

**Characteristic**	**Total (n=63)**	**OIC (n=31)**	**Non-OIC (n=32)**	***p*-value[a]**
Tramadol-equivalent dose (mg/day)				
Median	60.7	60.7	61.6	0.3804
Q1, Q3	46.4, 85.7	40.0, 85.7	50.0, 89.3	

OIC, opioid-induced constipation; Q, quartile.

[a]Mann-Whitney U test.

Supplementary Table 4. NRS score

Characteristic	Total (n=63)	OIC (n=31)	No OIC (n=32)	*p*-value[a]
NRS (0-10) at baseline				
n	61	29[b]	32	
Mean±SD	4.98±2.96	4.76±3.49	5.19±2.43	0.5838
NRS change (0-10) (days 8-14) from baseline				
n	61	29[b]	32	
Mean±SD	1.65±2.59	-1.66±3.21	1.64±1.92	0.9755

NRS, Numerical Rating Scale; OIC, opioid-induced constipation; SD, standard deviation.

[a]Unpaired t-test.

[b]Participants who answered at baseline.
